# Structural and Functional Organization of Visual Responses in the Inferior Olive of Larval Zebrafish

**DOI:** 10.1523/JNEUROSCI.2352-21.2023

**Published:** 2024-02-21

**Authors:** Rita Félix, Daniil A. Markov, Sabine L. Renninger, Ana Raquel Tomás, Alexandre Laborde, Megan R. Carey, Michael B. Orger, Ruben Portugues

**Affiliations:** ^1^Champalimaud Research, Champalimaud Centre for the Unknown, Lisbon 1400-038, Portugal; ^2^Max Planck Institute of Neurobiology, Sensorimotor Control Research Group, 82152 Martinsried, Germany; ^3^Institute of Neuroscience, Technical University of Munich, 80802 Munich, Germany; ^4^Munich Cluster of Systems Neurology (SyNergy), 81377 Munich, Germany

**Keywords:** calcium imaging, cerebellum, inferior olive, whole-field motion, zebrafish

## Abstract

The olivo-cerebellar system plays an important role in vertebrate sensorimotor control. Here, we investigate sensory representations in the inferior olive (IO) of larval zebrafish and their spatial organization. Using single-cell labeling of genetically identified IO neurons, we find that they can be divided into at least two distinct groups based on their spatial location, dendritic morphology, and axonal projection patterns. In the same genetically targeted population, we recorded calcium activity in response to a set of visual stimuli using two-photon imaging. We found that most IO neurons showed direction-selective and binocular responses to visual stimuli and that the functional properties were spatially organized within the IO. Light-sheet functional imaging that allowed for simultaneous activity recordings at the soma and axonal level revealed tight coupling between functional properties, soma location, and axonal projection patterns of IO neurons. Taken together, our results suggest that anatomically defined classes of IO neurons correspond to distinct functional types, and that topographic connections between IO and cerebellum contribute to organization of the cerebellum into distinct functional zones.

## Significance Statement

Using the transparent larval zebrafish, we systematically recorded the responses of inferior olive (IO) neurons to visual motion stimuli that drive optomotor and optokinetic behaviors. We find that most IO neurons respond selectively to one or more such stimuli. Individual neurons are tuned to specific directions of motion and different functional types are distributed nonuniformly in the IO. Furthermore, we were able to link the functional type of the IO neurons with their location, morphology, and projection patterns. This shows how topographically organized projections from the IO play an important role in channeling behaviorally relevant information to the cerebellum in zebrafish larvae.

## Introduction

The olivo-cerebellar system plays an important role in sensorimotor control and coordination in vertebrates. The inferior olive (IO) sends climbing fiber (CF) projections to the cerebellum, where each CF makes extensive excitatory synaptic connections with a single Purkinje cell in such a way that a single presynaptic action potential elicits a characteristic postsynaptic complex spike (Eccles et al., 1966). These complex spikes play a fundamental role in modulating Purkinje cell simple spikes and thus fine-tune cerebellar output (Marr, 1969; Albus, 1971; Ito et al., 1982).

Studies in mammals have identified a population of IO neurons that receives input from direction-selective cells in the accessory optic system, a visual pathway mediating the detection of optic flow (Soodak and Simpson, 1988). This population conveys visual sensory error signals to the cerebellum in the form of retinal slip, giving rise to complex spikes that are direction selective, and out of phase with simple-spike firing rate (Ito, 1982; Stone and Lisberger, 1990). In addition to direction selectivity, electrophysiology studies have found evidence of functional organization of CF projections. For instance, CFs carrying motion signals consistent with rotation about horizontal or vertical axes project to distinct zones of the cerebellar flocculus (Schonewille et al., 2006; Pakan et al., 2011). Moreover, in pigeons, cerebellar zones responding to different types of optic flow stimuli (Wylie and Frost, 1993; Wylie et al., 1993) have been shown to receive input from different regions of the IO (Craciun et al., 2018).

Recent calcium imaging of Purkinje cells in zebrafish larvae has revealed distinct areas of the cerebellum associated with motion stimuli that drive distinct visuomotor behaviors: the optomotor response (OMR), which drives the fish to swim and turn in the direction of visual motion, and the optokinetic reflex (OKR), which drives the eyes to track the direction of the rotation and make rapid resetting saccades in the opposite direction (Matsui et al., 2014; Knogler et al., 2019). Additionally, electrophysiological recordings from larval zebrafish Purkinje cells, located in different regions of the cerebellum, showed that complex spike responses can be grouped into different visual categories, related with changes in luminance and direction-selective translational or rotational motion (Knogler et al., 2019). Since translational and rotational motion stimuli drive different visually driven motor behaviors in larval zebrafish (Neuhauss et al., 1999), these results have led to the proposed separation of the zebrafish cerebellum into different behavioral modules. According to this model, activity in the medial cerebellum is associated with swimming and turning movements during the OMR, the medial–lateral cerebellum processes changes in luminance, and the lateral cerebellum is involved in eye and body coordination during the OKR (Matsui et al., 2014; Knogler et al., 2019). Distinct functional mapping across medial–lateral cerebellar regions in larval zebrafish is further supported by anatomical mapping of cerebellar outputs (Heap et al., 2013; Takeuchi et al., 2015; Kunst et al., 2019).

Taken together, these studies suggest that spatially segregated and functionally distinct Purkinje cells can differentially modulate various aspects of sensorimotor control and coordination. However, the extent to which a topographic organization is already present in the zebrafish IO and is fed forward by topography-preserving CF projections, or whether a different principle organizes the representations in the IO, is still unknown. This is partly because most functional studies have focused on Purkinje cell complex spikes as a proxy for IO activity, due to technical challenges associated with recording activity from IO neurons in behaving animals.

In this study, we take advantage of the larval zebrafish’s small, transparent brain in combination with genetic tools to investigate the IO, to better understand its structural and functional organization at a cellular and population level. We show that IO neurons can be divided into at least two distinct anatomical types based on their spatial location, dendritic morphology and axonal projection patterns. Furthermore, most IO neurons respond to visual stimuli that drive distinct behaviors in a direction-selective manner, with functional properties spatially organized within the IO. We describe both anatomical and functional segregation of IO neurons that can be associated with different cerebellar modules, relevant for visually driven behaviors such as the OMR and OKR.

## Materials and Methods

### Experimental design and statistical analysis

**Figure 1.**
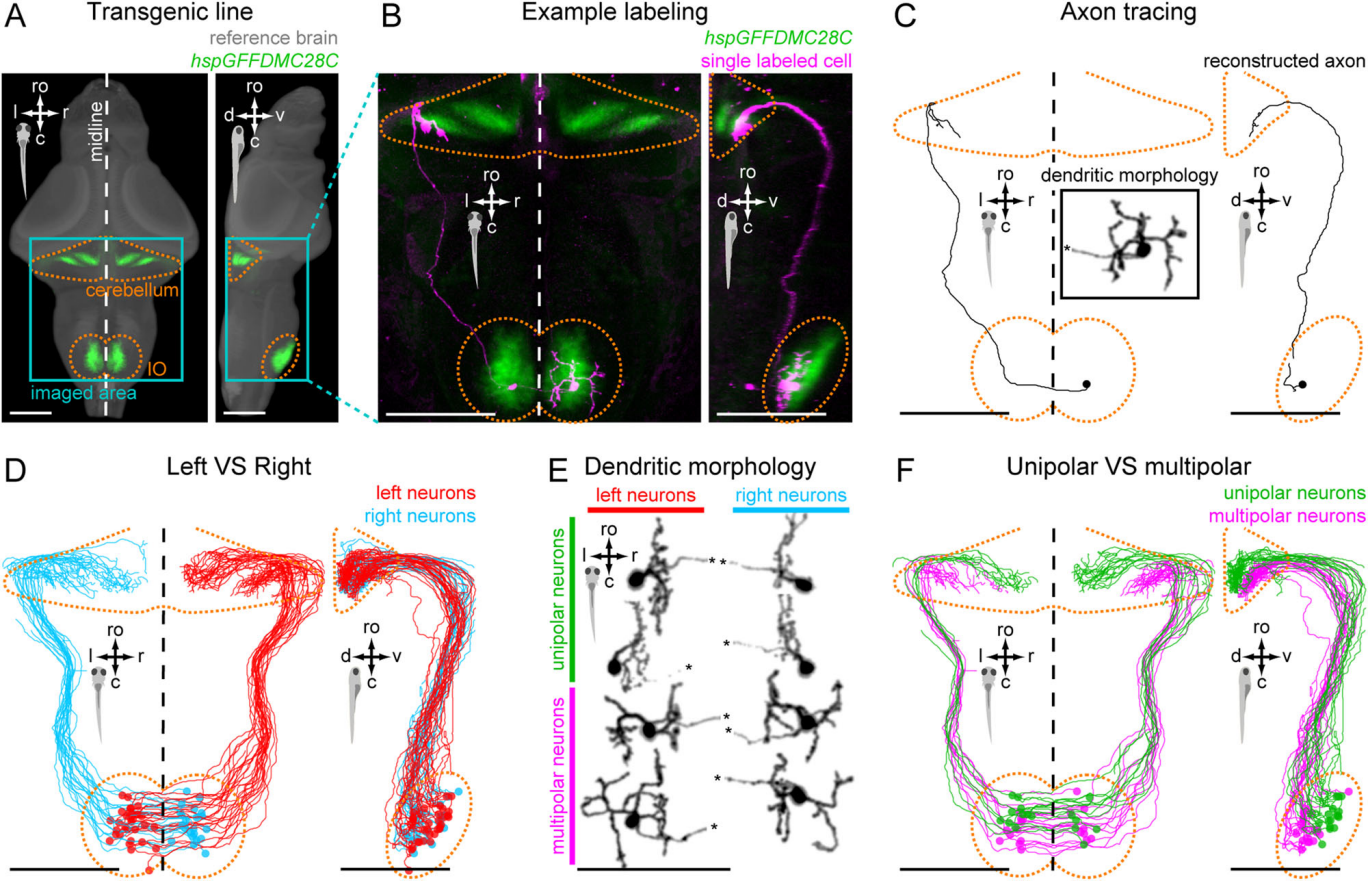
IO neurons can be divided into distinct morpho-anatomical types. ***A***, Dorsal and lateral views of the average expression of the *hspGFFDMC28C* line used in this study (green, *N* = 39 fish; line from Takeuchi et al., 2015) registered to a common reference larval zebrafish brain (gray), showing strong signal in the IO and in the CFs in the cerebellum. In this and subsequent panels: ro, rostral direction; l, left; r, right; c, caudal; d, dorsal; v, ventral; scale bars, 100 µm; vertical dashed lines indicate the midline of the brain. Teal rectangle outlines the area shown in B, C, D, and F. ***B***, Example of a single labeled IO neuron (magenta). ***C***, Axon reconstruction of that neuron. The inset shows its dendritic morphology, and the asterisk indicates its axon. ***D***, Axon reconstruction of all labeled IO neurons (*N* = 53 neurons from 39 larvae) color coded by soma location (red, left IO, teal, right IO), showing that IO neurons project contralaterally. ***E***, Examples of IO neurons that were divided into two morphological classes: unipolar neurons (green) that have a single dendritic tree arborized along the midline, and multipolar neurons (magenta) that have bi- or tri-polar dendritic trees. Asterisks indicate axons. For the complete dataset (*N* = 16 unipolar, 19 multipolar and 18 ambiguous neurons) see Figure 1-1. ***F***, Axon reconstruction of all unipolar (green) and multipolar neurons (magenta), showing that the morphological type of a neuron is predictive of its projection pattern and its location within the IO.

**Figure 2.**
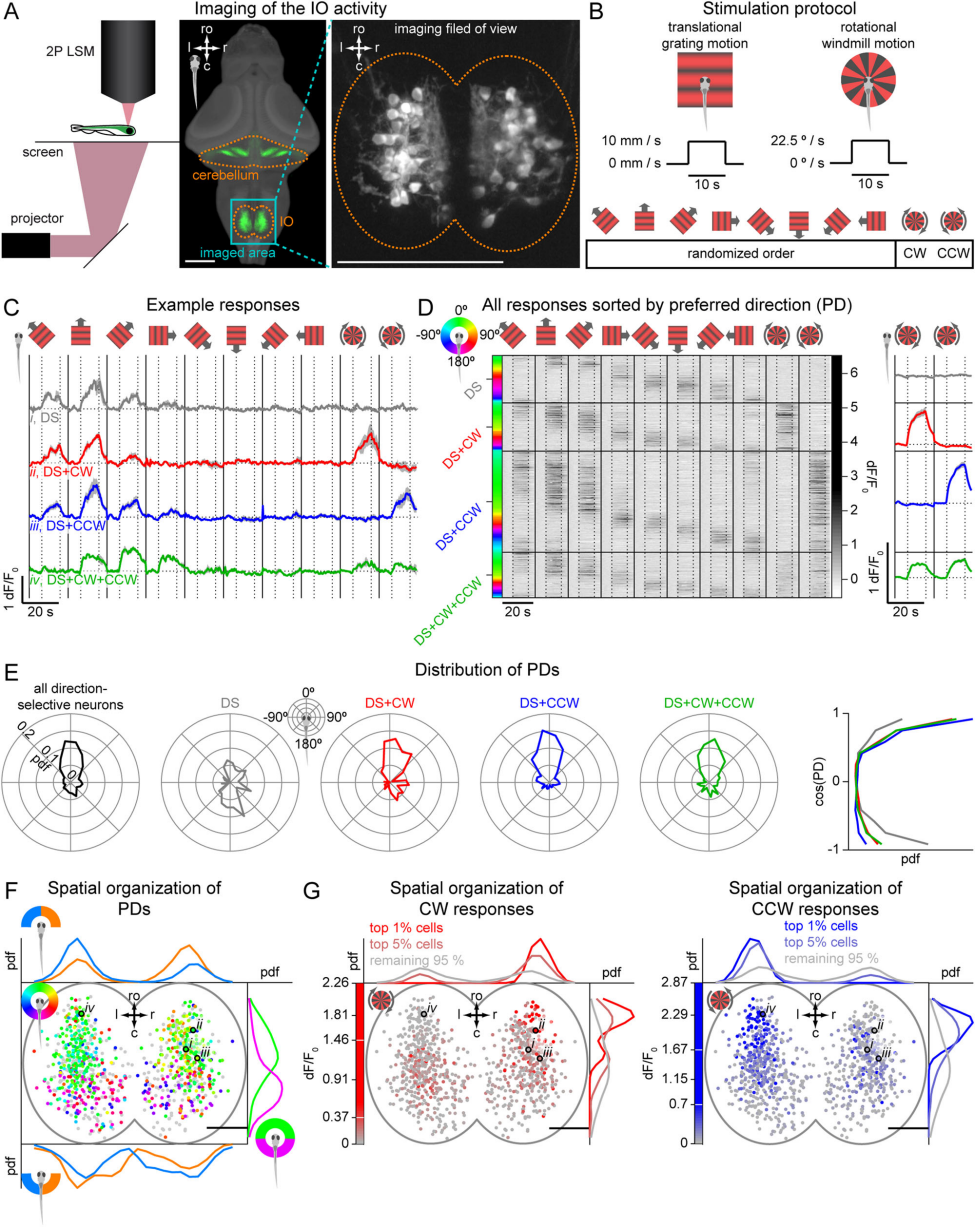
The majority of IO neurons are sensitive to translational and rotational motion, direction-selective and spatially organized. ***A***, Left, two-photon laser-scanning microscope (2P LSM) used for calcium imaging. Middle, same as Figure 1*A*; ro, rostral direction; l, left; r, right; c, caudal; scale bars, 100 µm. Teal rectangle outlines the imaged area. Right, maximum intensity *z*-projection of the anatomy stack from an example fish, showing a typical imaging field of view with individual IO neurons. ***B***, Stimulation protocol. Fish were presented with translational and rotational motion, with each stimulus lasting 21.4 s (6 s stationary, 10 s moving, 5.4 s stationary). Translational gratings moved at 10 mm/s and rotational windmill at 22.5 °/s. For each imaging plane, we presented translational gratings in 8 different directions in a randomized order, followed by clockwise (CW) and counter-clockwise (CCW) rotational motion. ***C***, Average responses of four forward-selective example neurons to translational and rotational motion. DS, direction-selective neurons responding exclusively to translation motion, DS + CW or DS + CCW, direction-selective neurons that also responded to CW or CCW rotation, DS + CW + CCW, direction-selective neurons that also responded to rotation in both directions. Shadows represent SEM across repetitions. Horizontal dotted lines represent each neuron baseline. In C and D, vertical dotted lines separate stationary and moving periods of the stimulus, vertical solid lines separate visual stimuli. ***D***, Left, raster plot for all direction-selective neurons (*N* = 608 neurons from 12 fish), grouped by response types and sorted by PD (coded by the circular color wheel). Each row represents a neuron’s average response to the ten stimuli shown on top. Right, average response of all neurons in each group to CW and CCW stimuli. Shadows represent SEM across neurons. ***E***, Left, distribution of PDs of all direction-selective neurons and of the 4 different groups of neurons independently. 0° represents forward, 90° rightward, 180° backward and −90° leftward directions. Right, probability distribution of the cosine of PD for each of the four groups. ***F***, Spatial distribution of PDs within the IO (*N* = 967 active neurons, including 608 direction-selective ones, from 12 fish). Each dot represents a neuron color-coded for PD as represented in the color wheel, or gray for not direction-selective neurons. Small italic roman numbers indicate the location of neurons shown in C. In F and G: ro, rostral direction; l, left; r, right; c, caudal; scale bars, 25 µm. Green and magenta curves show the rostral–caudal distribution of forward- and backward-preferring neurons respectively, separated according to the sign of the PD cosine. Blue and orange curves show the left-right distribution of left- and right-preferring neurons respectively, separated into forward-preferring (top) and backward-preferring (bottom) groups. ***G***, Spatial distribution of rotation sensitivity within the IO. Each dot represents a neuron color-coded for its response to CW (red) and CCW (blue) stimuli. White lines on the color bars denote 99^th^ and 95^th^ percentiles, used for presenting the spatial distributions of top 1%, top 5%, and remaining 95% active neurons.

**Figure 3.**
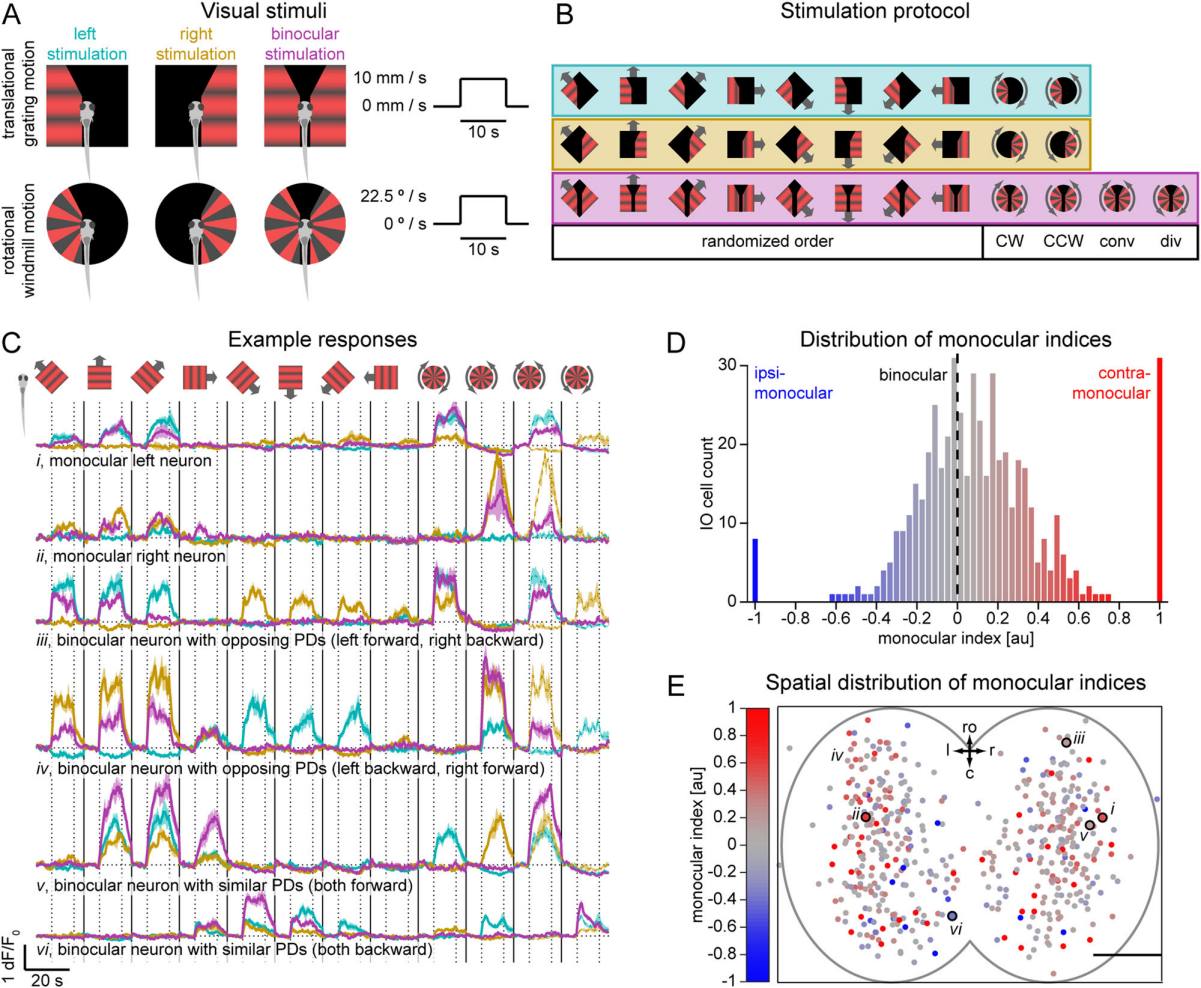
The majority of IO neurons receive input from both eyes with a contralateral bias. ***A***, Monocular visual stimuli. Fish were presented with translational and rotational motion, with each stimulus lasting 21.4 s (6 s stationary, 10 s moving, 5.4 s stationary). Translational gratings moved at 10 mm/s and rotational windmill at 22.5°/s. Each stimulus was presented three times per imaging plane: to the left eye, to the right eye, and binocularly. To avoid contralateral contamination during monocular stimulation, the two visual fields were separated by a vertical 0.5 mm black patch that was positioned below the fish body. In addition, we had a 55° cut-off in front of the fish (27.5° in each eye) to prevent stimulation of the eyes’ binocular zone. ***B***, The trial structure was the same as in the binocular stimulation experiment (translational gratings in 8 different directions in a randomized order, followed by clockwise (CW) and counter-clockwise (CCW) rotational motion). To minimize possible contribution of light onset/offset to the responses, monocular and binocular stimulation were performed in groups: left, followed by right and finally binocular stimulation. Binocular stimulation block also included converging (conv) and diverging (div) rotational motion. ***C***, Examples of neurons’ responses to monocular stimulation of the left (cyan) and right (yellow) visual fields and to binocular stimulation (magenta). Vertical dotted lines separate stationary and moving periods of the stimulus. Horizontal dotted lines represent each neuron baseline. Vertical solid lines separate different visual stimuli. For comparison purposes, all example neurons monocular CW and CCW responses are repeated in binocular convergence and divergence stimuli (dashed lines). Shadows represent SEM across repetitions. ***D***, Distribution of neurons’ monocular index. Monocular bias is color coded with a red (contra) through gray (binocular) to blue (ipsi) gradient. Dashed line indicates unbiased binocular neurons. Note that the distribution is shifted to the right, indicating that IO neurons are in general more sensitive to contralateral stimulation. ***E***, Spatial distribution of monocular bias within the IO, color coded as histogram in D; ro, rostral direction; l, left; r, right; c, caudal; scale bar, 25 µm. Small italic roman numbers indicate the location of neurons shown in C. *N* = 518 neurons (of which 511 were active) from six fish.

**Figure 4.**
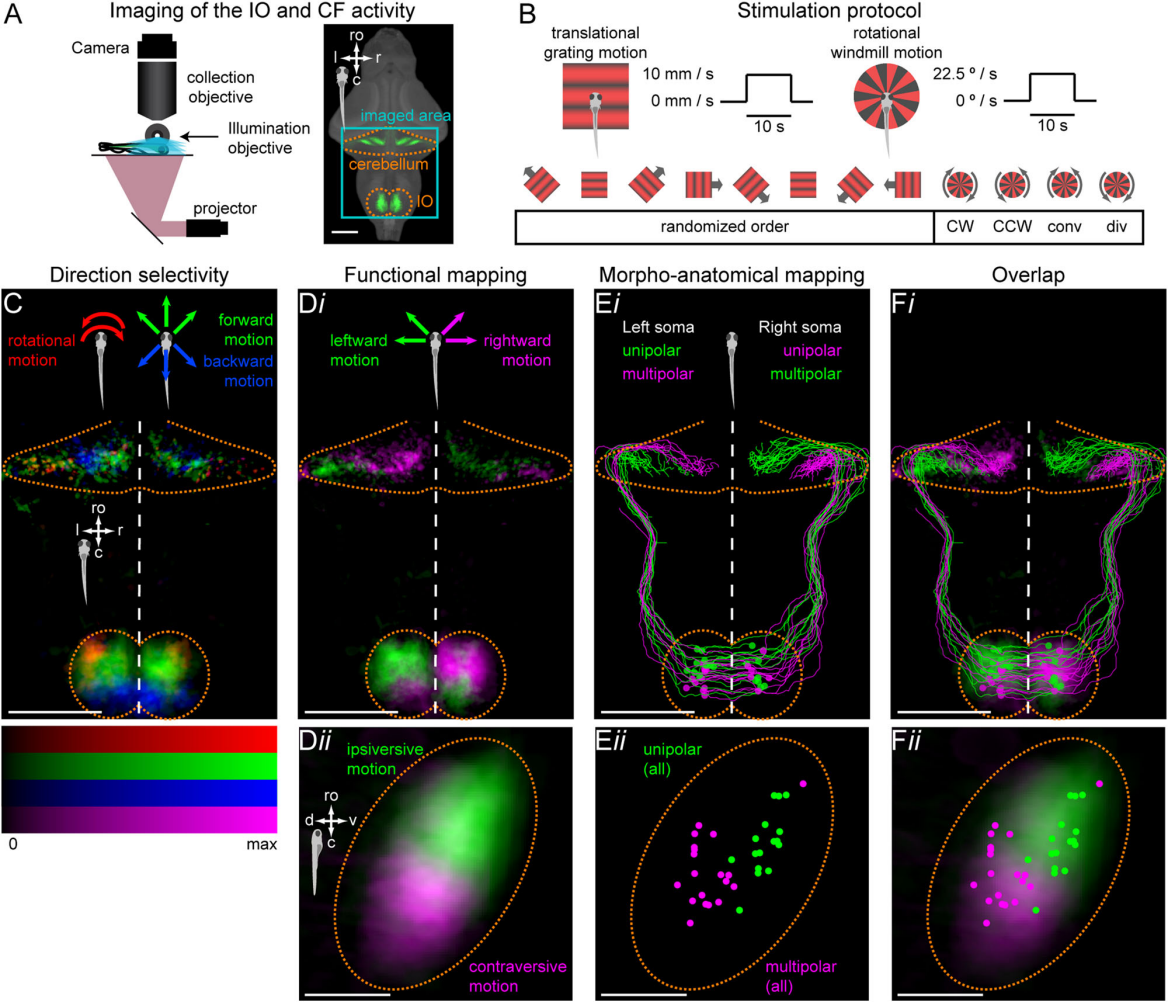
Functional organization of the IO maps onto its morpho-anatomical organization. ***A***, Left, light-sheet microscope used for fast volumetric calcium imaging. Right, same as Figure 1*A*. Teal rectangle outlines the imaged area. ***B***, Stimulation protocol. The trial structure was the same as in the binocular stimulation experiment [translational gratings in 8 different directions in a randomized order, followed by clockwise (CW) and counter-clockwise (CCW) rotational motion]. Additionally, we included converging (conv) and diverging (div) rotational motion. Each stimulus lasted 21 s (6 s stationary, 10 s moving, 5 s stationary). Translational gratings moved at 10 mm/s and rotational windmill at 22.5 °/s. For each fish, we presented this stimulus set five times. ***C***, Max *z*-projection of the distribution of active voxels categorized as forward selective (green), backward selective (blue) or rotation selective (red), averaged across fish. ***D i***, Max *z*-projections of the distribution of active voxels categorized as left selective (green) or right selective (magenta) in the entire imaging field of view. ***D ii***, Max lateral projection of the distribution of active voxels selective for ipsiversive motion (green) and for contraversive motion (magenta) within the IO. ***E i***, Max *z*-projection patterns of unipolar and multipolar neurons from Figure 1*F*, color-coded depending on their morphological type and left-right location within the IO. ***E ii***, Lateral projection of the location of unipolar (green) and multipolar neurons (magenta) within the IO. ***F***, Overlay of D and E. Note that the distribution of active voxels in the IO in the light-sheet imaging data includes signals not only from cell somata but also from surrounding neuropil, which accounts for the more lateral spread compared to the soma distribution. See Table 1 for quantification of the overlap. *N* = 28 fish; ro, rostral direction; l, left; r, right; c, caudal; v, ventral; d, dorsal; scale bars, 100 µm.

This study was aimed to characterize morpho-anatomical and functional properties of the IO neurons in larval zebrafish in order to understand whether the topological organization described in the zebrafish cerebellum is already present in its presynaptic source, the IO. To this end, we designed the following four experiments:

1. Experiment 1: Morpho-anatomical characterization of the IO neurons (Fig. 1). In this experiment we used 39 zebrafish larvae to label 53 individual IO neurons and to characterize their dendritic morphology, anatomical location within the IO, and projection patterns within the cerebellum.
2. Experiment 2: Characterization of the IO neuronal activity in response to whole-field translational and rotational visual motion (Fig. 2). Such stimuli were chosen because they are known to induce distinct behavioral responses in larval zebrafish and are associated with activity in distinct anatomical regions in the cerebellum (Matsui et al., 2014). In this experiment, we recorded activity of 1,106 IO neurons from 12 animals.
3. Experiment 3: Characterization of the IO neuronal activity in response to monocular stimulation (Fig. 3). In this experiment, we aimed to understand how (if at all) the responses observed in Experiment 2 result from binocular integration of monocular inputs. To this end, we recorded activity of 518 IO neurons from six animals.
4. Experiment 4: Linking the morpho-anatomical and functional organization of the IO (Fig. 4). To this end, we recorded binocular responses from 28 larvae not only from the somata of the IO neurons but also from their axon terminals within the cerebellum using light-sheet microscopy. This approach allowed us to directly link the morpho-anatomical and functional properties of the IO neurons with the functionally compartmentalized organization of the zebrafish cerebellum.

10.1523/JNEUROSCI.2352-21.2023.f1-1 Figure 1-1 **The complete dataset of single-labeled IO neurons** **A**. Dendritic morphology of all labeled IO neurons divided by their morphological class (green, 16 unipolar neurons, magenta, 19 multipolar neurons, black, 18 ambiguous neurons). Asterisks indicate axons. **B**. Axon reconstruction of all labeled neurons; ro, rostral direction, l, left, r, right, c, caudal, d, dorsal, v, ventral; scale bars, 100 µm. Download Figure 1-1, TIF file.

10.1523/JNEUROSCI.2352-21.2023.f3-1 Figure 3-1 **Rotation-sensitive IO neurons often receive inputs with opposing PDs and occupy distinct regions within the IO** **A**. Left, maximal response to rotational stimuli as a function of the absolute monocular index of each neuron (left) and of absolute difference in the PDs of the left and right visual fields (right). **B**. Binocular PD as a function of left and right eye PDs, color-coded as in color wheel on top. Gray dots represent non direction-selective cells. In B, C, and D: small italic roman numbers indicate example neurons shown in Figure 23. **C**. Responses to binocular CW rotation (left) and CCW rotation (right) as a function of left and right PDs. **D**. Spatial organization of the IO neurons, color-coded based on their monocular PDs. Shown in red and blue are neurons with opposing PDs (upper left and bottom right quadrants in B and C). Green and magenta indicate neurons with similar PDs (green: forward monocular PDs, bottom left quadrant; magenta: backward PDs, upper right quadrants). Ro, rostral direction, l, left, r, right, c, caudal; scale bar, 25 µm. Small italic roman numbers indicate the location of neurons shown in C. *N* = 518 neurons (of which 511 were active) from 6 fish. Download Figure 3-1, TIF file.

10.1523/JNEUROSCI.2352-21.2023.f4-1 Figure 4-1 **Functional mapping based on responses to rotational, forward and backward motion does not correspond to the morpho-anatomical organization of the IO** **A**. Max projections of the distribution of active voxels color-coded using different functional axes: **A *i***, rotational-selective (green) VS backward-selective voxels (magenta); **A *ii***, forward-selective (green) VS backward-selective voxels (magenta). **A *iii*** is reproduced from Figure 4D for comparison to other functional mappings. Images on top show max z-projections of the entire imaging field of view that included IO and CFs, bottom images show zoomed-in lateral projections zoomed into the IO. **B**. Overlap of respective functional maps with morpho-anatomical mapping within the IO. Each dot represents one unipolar or multipolar neuron from Figure 1. See Table 1 for quantification of the overlap. *N* = 28 fish; ro, rostral direction, l, left, r, right, c, caudal, v, ventral, d, dorsal; scale bars, 100 µm. Download Figure 4-1, TIF file.

Experiments 1–3 allowed us to formulate three specific hypotheses for how functional organization might be related to morpho-anatomical organization. In Experiment 4, we specifically test these hypotheses using a bootstrapping procedure described in the Materials and Methods section, Quantification of overlap (see below). The significance level was set to 5% and *p*-value thresholds were Bonferroni-corrected for six comparisons (3 hypotheses times two brain regions: IO and cerebellum). We used one-tailed alternatives because results of Experiments 1–3 allowed us to generate specific expectations of the relationship between function and anatomy.

Experiments were performed in accordance with the European Directive 2010/63/EU and approved by the Champalimaud Ethics Committee and the Portuguese Direcção Geral Veterinária (Ref. No. 019774) and approved protocols set by the Max Planck Society and the Regierung von Oberbayern (TVA 55-2-1-54-2532-82-2016).

All data and code used in this study can be made available upon request.

### Experimental animals

#### Zebrafish husbandry

All experiments were performed on larval zebrafish (*Danio rerio*) at 6–7 d post-fertilization (dpf), with the exception of single-cell electroporation (see below) that was performed at 5–6 dpf. The sex of the animals could not be determined at this early developmental stage.

Zebrafish breeding and maintenance were performed under standard conditions (Westerfield, 2007; Martins et al., 2016). Both adult fish and larvae were maintained at 28°C on a 14/10 h light/dark cycle. Adult zebrafish were housed in a zebrafish facility system with constantly recirculating water with about 10% daily water exchange. Fertilized embryos were collected in the morning and kept in 94 mm Petri dishes at a density of 20 animals per dish in E3 medium with daily water exchange, unless otherwise specified.

#### Transgenic lines

All experiments were performed using a transgenic *hspGFFDMC28C* driver line (r*k8Tg*; Takeuchi et al., 2015), expressing a modified version of Gal4 mainly in the IO neurons (Fig. 1*A*). Animals were also homozygous for the *nacre* mutation, which introduces a deficiency in the *mitfa* gene involved in development of skin melanophores (Lister et al., 1999), thereby allowing for noninvasive brain imaging. The UAS reporter gene differed depending on the experiment.

For single-cell electroporation, we used the incross of *hspGFFDMC28C*; *UAS:mCherry* to inject plasmid DNA *pCS2-GAP43-GFP* construct (kindly provided by Isaac Bianco).

For sparse single-cell genetic labeling, we outcrossed *hspGFFDMC28C*; *UAS:GFP* to *UAS:epNtr-tagRFP* reporter line (line mpn123 generated by Miguel Fernandes and Herwig Baier at MPI Neurobiology).

For functional imaging experiments, we used the offspring of an incross of *hspGFFDMC28C*; *UAS:GCaMP6fEF05* (*ccu2Tg*) for IO-specific expression of the calcium indicator GCaMP6fEF05. This modified version of GCaMP6f (Chen et al., 2013) was made by making the mutations *D397N/G398A/N399D* (Sun et al., 2013) in the CaM domain of GCaMP6f. This version reports activity in zebrafish neurons with better signal to noise ratio than GCaMP6f, while maintaining its fast dynamics in comparison to GCaMP6 s (Ostrovsky, Renninger et al., in prep.).

### Experiment 1: morpho-anatomical characterization of the IO neurons

The first aim of this study was to characterize the morpho-anatomical properties of the IO neurons in larval zebrafish by the means of single-cell labeling (Fig. 1). Labeling of individual IO cells was achieved by either single-cell electroporation or by sparse genetic labeling.

#### Single-cell electroporation

5–6 dpf *hspGFFDMC28C*; *UAS:mCherry* larvae were embedded in 1.5% low melting point agarose, anesthetized in bath-applied solution of MS-222 (tricaine) at a concentration of 0.16 g/L in Danieau’s solution (58 mM NaCl, 0.7 mM KCl, 0.4 mM MgSO_4_, 0.6 mM Ca(NO_3_)_2_, 5 mM HEPES buffer), and IO neurons were electroporated under a confocal microscope (LSM 780, Carl Zeiss) as described previously (Tawk et al., 2009). Briefly, a fine borosilicate glass electrode with filament (final tip diameter ∼1 µm) was filled with plasmid DNA *pCS2-GAP43-GFP* construct (kindly provided by Isaac Bianco) at a concentration of ∼1 µg/µl in distilled water and manipulated through the tissue to a target mCherry-positive IO neuron using a micromanipulator (Sutter Instruments). 1–3 square trains of electric pulses with a frequency of 200 Hz, duration of 1 s, and magnitude of 20–30 V were applied to inject DNA constructs into a single neuron using an Axoporator 800A (Molecular Devices). After electroporation, dishes with embedded larvae were gently washed with Danieau’s solution three times to wash out the tricaine. After washing, larvae were released from the agarose and allowed to recover in Danieau’s solution overnight.

#### Sparse genetic labeling

Sparse genetic labeling of individual IO neurons was achieved by outcrossing the *hspGFFDMC28C*; *UAS:GFP* to a *UAS:epNtr-tagRFP* reporter line. The offspring of such an outcross typically had very sparse expression of RFP, often in only one or two IO neurons, which was ideal for neuronal tracing.

#### Confocal imaging

To image the labeling results, 6–7 dpf larvae were anesthetized using tricaine and embedded in 1.5% low melting point agarose. Labeled neurons were imaged using a confocal microscope (LSM 780, Carl Zeiss). For each successfully labeled larva, two *z*-stacks were acquired: one for visualizing the cell body and dendritic arbors at higher magnification (Fig. 1*E* and Fig. 1-1*A*), and another to capture the whole span of axonal projections at lower magnification. The second stack was acquired in two channels: a single-cell channel (GFP for the electroporated larvae and RFP for larvae with sparse genetic labeling) and an anatomical reference channel containing dense signal from the majority of the IO neurons and their projections (mCherry and GFP, respectively) (Fig. 1*B*). The latter channel was used for anatomical registration of the data to a standard reference brain (see Anatomical registration).

#### Tracing

After anatomical registration, axonal projections of labeled IO neurons were traced and reconstructed using the “Simple Neurite Tracer” FIJI plugin (Longair et al., 2011; Schindelin et al., 2012) (Fig. 1*C*). All animals, where IO morphology and projection pattern could not be clearly and unambiguously traced (due to too dense labeling or low signal), were not analyzed. After exclusion, 39 animals were used to label 53 individual IO neurons.

### Experiment 2: responses to whole-field visual stimulation

The next aim was to characterize the activity of the IO neurons in response to whole-field translational and rotational visual motion using two-photon calcium imaging in awake animals (Fig. 2). In total, we recorded activity of 1,106 IO neurons from 12 larvae expressing an enhanced version of GCaMP6f specifically in the IO neurons (see Transgenic lines for more detail).

#### Two-photon functional imaging

Functional imaging experiments were conducted on head-restrained preparations of 6–7 dpf zebrafish larvae (Portugues and Engert, 2011). Each larva was embedded in 2% low melting point agarose (Invitrogen, Thermo Fisher Scientific) in a 35 mm Petri dish with a Sylgard 184 base (Dow Corning). After allowing the agarose to set, the dish was filled with E3 medium, and the agarose around the tail and eyes was removed to allow for tail and eye movements that were used as a readout of behavior. Note that, in this study, the recorded behavioral traces were not analyzed.

The dish with the embedded larva was then placed onto a light-diffusing screen, located 5 mm below the larva, and imaged on a custom-built two-photon microscope (Fig. 2*A*, left).

A Ti-Sapphire laser (Coherent Chameleon) tuned to 950 nm wavelength was used for excitation and custom-written Labview software was used to control the microscope and to capture image data. Larval brains were systematically imaged from dorsal to ventral, in 2 µm *z*-steps, at approximately 3 Hz (345.6 ms/frame). In each fish, we imaged 30–40 planes which corresponded to 60–80 µm that covered the entire IO volume (Fig. 2*A*, right).

In addition, two infrared LEDs (850 nm wavelength) were angled between the imaging objective and the petri dish to illuminate the fish and allow the tracking of the tail. Behavior was recorded using a Mikrotron EoSens (MC1362) high-speed camera and a National Instruments frame grabber (PCIe-1433) (not shown in Fig. 2*A*). Tail tracking was performed at 700 Hz and eye-tracking at 100 Hz using a custom-written software in C#.

#### Binocular visual stimuli

For visual stimulation, we used a custom-written rendering engine that uses fragment shaders in OpenGL to draw visual stimuli in synchronization with two-photon imaging software. Visual stimuli were projected from below onto a flat screen at 60 Hz using a Optoma ML750e LED projector and a red colored glass long-pass filter (Thorlabs FGL590) and Texas Red bandpass emission filter (Thorlabs MF630-69) to allow for simultaneous imaging and visual stimulation.

Visual stimuli were projected from below onto a flat diffusing screen, located 5 mm below the fish and centered under its head. The stimulus set consisted of 10 stimuli per imaging plane: whole-field sine black-and-red gratings with a 10 mm spatial period moving in eight directions at 10 mm/s in a randomized order, followed by whole-field square black-and-red windmill stimulus rotating at 22.5°/s clockwise (CW) and then counterclockwise (CCW) (Fig. 2*B*). Each stimulus lasted 21.4 s (6 s stationary, 10 s moving, 5.4 s stationary), which corresponded to 62 imaging frames. After presenting the complete stimulus set, the imaging plane was moved 2 µm ventrally and the set of stimuli was repeated.

In our study, we show visual stimuli on a screen below the fish, as is the common practice in larval zebrafish. These stimuli have been well characterized in the context of optomotor and optokinetic behavior but do not allow mapping of visual responses originating from the upper visual field, in contrast to studies in other species which have used planetarium style surround stimulation. In a recent study in zebrafish, motion noise stimuli were presented on a vertical cylindrical screen to map the spatial receptive fields of optic flow responses in the pretectum of the zebrafish larva (Zhang et al., 2022). It was found that, of the neurons with bimodal receptive fields that could distinguish translational and rotational motion, a large majority was sensitive to translation in the horizontal plane, and horizontal rotations, consistent with the stimuli used in our study.

#### Image registration

Image processing and analysis was performed automatically using custom-written MATLAB code (MathWorks). To correct for motion artifacts and possible drifting of the animals inside the agarose, acquired frames were first aligned within a plane then across planes similarly to (Portugues et al., 2014). Any experiments during which the fish drifted significantly in *z* were stopped and the data discarded. If a frame could not be aligned to adjacent ones due to strong movement artifacts, this frame was excluded from the analysis. To generate anatomy stacks for each larva, all aligned frames within each plane were averaged. We then registered the anatomy stacks of individual fish to a common IO reference (see Anatomical registration).

#### Manual cell annotation

IO neurons were selected using a semi-manual 3D ROI selection tool custom written in MATLAB (MathWorks). This tool loaded an anatomy stack and allowed for ROI selection across planes. Given the anatomy of IO neurons, we could select with a high degree of certainty an ROI that corresponded to a single neuron. The selection was based on selecting the center of a cell soma, defining its maximum pixel size and automatically selecting similar intensities around the center in 3D that could be manually refined to better match the cell’s morphology. Since the scanning was performed plane by plane, the number of trials for which the activity of a given neuron was sampled depended on the number of planes covered by that neuron. As a result, different neurons might have different numbers of repetitions. The centroid of each neuron was used for IO spatial distribution analysis.

#### Analysis of neuronal responses to binocular stimulation

After annotating IO neurons, we extracted their activity by averaging the fluorescence traces of all pixels composing a given neuron at a given plane. Fluorescence signals were then converted to dF/*F*_0_. For each stimulus, dF/*F*_0_ was calculated for every frame as the fluorescence signal (*F*) normalized by the baseline: the average signal recorded during the first 6-s stationary period of that stimulus (*F*_0_).

The response of a given neuron to a given stimulus was defined as the mean dF/*F*_0_ during the 10-s moving period of that stimulus, averaged across repetitions. A neuron was considered as responding to a given stimulus, if its response exceeded the mean dF/*F*_0_ computed for the 6-s stationary baseline period of that stimulus plus 2 standard deviations. A neuron that responded to at least one stimulus from the set was referred to as active.

Direction selectivity was quantified by a normalized direction selectivity index (DSI), calculated as the vector sum of mean responses to translational stimuli moving at all eight directions divided by the sum of the responses. This means that a neuron that responded to only one translational stimulus would have a DSI of 1 and an active neuron that responded equally to all directions would have a DSI of 0. Preferred direction (PD) corresponded to the DSI angle.

Direction-selective neurons were defined using the following bootstrapping procedure. We first formulated a null hypothesis for each neuron, that it was not direction selective and an alternative hypothesis that it was. If the null hypothesis is true, random shuffling of the responses should not change the DSI. We therefore performed random shuffling of the responses 1,000 times and computed the null distribution of DSI’s under the assumption that the null hypothesis was correct. If the actual DSI was greater than the 95th percentile of the null distribution, this neuron was considered direction selective.

### Experiment 3: responses to monocular visual stimulation

The next aim was to characterize the activity of the IO neurons in response not only to whole-field moving stimuli but also to stimuli presented only to one eye (Fig. 3). This was achieved by using the same method as described in the previous section *(*see Experiment 2: responses to whole-field visual stimulation). The only difference concerned the stimulus set presented during the functional imaging, and how these stimuli were presented. In total, we recorded activity of 518 IO neurons from six larvae.

#### Monocular visual stimuli

To avoid light contamination of either visual field during monocular stimulation, the two visual fields were separated by a 1 cm width black patch that was positioned below the fish body (Bianco et al., 2011). In addition, we had a 55° cut-off in front of the fish (27.5° in each eye) to prevent stimulation of the fish’s binocular field (Fig. 3*A*).

The temporal structure of each stimulus presentation was preserved from Experiment 2: 6 s stationary, 10 s moving, and 5.4 s stationary. However, each stimulus was presented three times per plane (instead of just one for Experiment 2): to the left eye, to the right eye, and binocularly. To avoid possible contribution to the responses of switching the stimulus on and off, monocular and binocular stimulation were performed in blocks (left, followed by right and finally binocular stimulation). In addition, the first stimulus within each block was presented twice, and the first repetition of the first stimulus was discarded. Similarly to Experiment 2, each stimulation block consisted of the presentation of translational gratings in eight different directions in a randomized order, followed by CW and CCW rotational stimulus; and additional converging and diverging rotational motions were added to the binocular stimulation block (Fig. 3*B*).

#### Analysis of neuronal responses to monocular stimulation

Imaging data analysis was performed similarly to Experiment 2. Definitions and metrics used for Experiment 2 were maintained (responding, active, direction-selective neurons, PD, DSI) but applied independently to left, right, and binocular stimulation blocks. Thus, a neuron can technically be defined, for example, as direction selective and forward preferring during left but not during right stimulation.

In addition, we computed the monocular index for each neuron by subtracting responses to stimuli presented to the ipsilateral eye from those presented to the contralateral eye (averaged across repetitions and directions) and dividing by the sum of these responses. This index can range from −1 (ipsi-monocular neuron that responds only to ipsilateral stimulation) through 0 (binocular neuron that responds equally to ipsi- and contralateral stimulation) to 1 (contra-monocular neuron that responds only to contralateral stimulation).

### Experiment 4: link between morpho-anatomical and functional organization of the IO

The last aim of this study was to determine whether the morpho-anatomical organization observed in Experiment 1 is consistent with the functional organization observed in Experiments 2 and 3 (Fig. 4). To this end, we performed functional light-sheet imaging as the animals were presented with whole-filed visual motion. This allowed us to simultaneously record the activity not only from the IO cell somata but also from their axon terminals within the cerebellum, and to relate observed functional organization to morpho-anatomical organization of the IO and to previously described functional compartmentalization of the zebrafish cerebellum.

#### Light-sheet functional imaging

Light-sheet functional imaging was conducted using head-restrained preparations of 6–7 dpf zebrafish larvae (Portugues and Engert, 2011). To avoid potential movement artifacts, each larva was paralyzed in bath-applied ɑ-bungarotoxin 2 mg/ml for 5–10 s, and embedded in 1.6% low melting point agarose (Invitrogen, Thermo Fisher Scientific) in a 35 mm Petri dish with a Sylgard 184 base (Dow Corning). After allowing the agarose to set, the dish was filled with E3 medium and the agarose around the eyes was removed to avoid light scattering. Each petri dish was cut and fit with a cover slip window that was systematically positioned on the left side of the fish. This allowed for unperturbed entry of the focused light sheet laterally onto the fish. The dish was placed onto a light-diffusing screen and imaged on a custom-built light-sheet microscope (Fig. 4*A*).

We used a blue excitation laser (MBL-FN-473, 473 nm, 200 mW, Changchun New Industries Optoelectronics Technology), controlled by an acousto-optic modulator (MTS110-A3-VIS, AA optoelectronics) that allowed for rapid changes in light-sheet power. The beam passed onto a first 1D galvo mirror (GVS011, Thorlabs) which scanned horizontally in order to create a sheet of light. We used a pair of lenses (AC254-100-A-ML, Thorlabs) to focus the light sheet onto a second 1D galvo mirror that allowed for the scanning of the light sheet vertically through the fish, and illuminate a series of optical slices to perform volumetric imaging. A 1D line diffuser was added in the pathway of the light sheet to reduce horizontal striping in the image due to shadowing of the light sheet by skin structures or blood vessels (Taylor et al., 2018). Fluorescence light was collected with a 20× water immersion objective (XLUMPlanFLN 20×/1.00 W) and two 525 nm bandpass filters were used to exclude any nongreen light from being detected by the camera (ORCA-flash 4.0, Hamamatsu). In order to image the illuminated plane as vertical scanning was performed and to keep it in focus, the objective was mounted on a Piezo stage (Piezosystem Jena) whose motion was synchronized with the second galvo. The lateral pixel size was measured to be 0.65 µm. Fish brains (*N* = 28 fish) were imaged at 100 Hz in volumes of 44 planes (2.3 Hz per volume) that covered an 220 µm square. A custom-written GUI developed by José Lima and Lucas Martins was used to control the microscope and to capture image data.

#### Visual stimuli of Experiment 4

Visual stimuli were projected from below onto a flat screen using a laser projector (SHOWWX+ Laser Pico Projector, MicroVision), and centered under the fish head. The screen was positioned roughly 5 mm from the embedded larva, as was done in previous studies (Portugues and Engert 2011; Severi et al., 2014).

The stimulus set was similar to that described for Experiment 2. It consisted of 12 whole-field stimuli: sine black-and-red gratings with a 10 mm spatial period moving in eight directions at 10 mm/s in a randomized order, followed by whole-field square black-and-red windmill stimulus rotating at 22.5 °/s at CW, CCW, converging and diverging direction (Fig. 4*B*). Each stimulus was presented for 21 s (6 s stationary, 10 s moving, 5 s stationary). This set of stimuli was presented five times, with translational directions being randomized in each repetition. Before the experimental protocol started, fish were habituated to laser scanning for 5 min. A single green frame, which bled through the filters sufficiently to be detected by the camera, was presented in the beginning and end of the experimental protocol, to allow imaging data and visual stimulus synchronization.

#### Analysis of light-sheet data

Image analysis of light-sheet data was performed with MATLAB (MathWorks). Volumetrically acquired light-sheet data was first corrected for rigid translational drift over the course of the experiment using the MATLAB *imregtform* ,nd. Any experiments during which the fish drifted significantly in *z* were discarded. After exclusion, the dataset consisted of 28 larvae. We then registered the anatomy of individual fish to a common reference brain (see Anatomical registration).

For each stimulus, mean response (dF/*F*_0_) was calculated by averaging frames during the moving period of the stimulus (*F*), and normalizing by the baseline (*F*_0_), which was calculated by averaging the frames when the stimulus was stationary. Each stimulus response was then averaged across the five repetitions (trials). This allowed us to create stack average activity maps for each stimulus for all fish.

For subsequent voxelwise analysis, we first selected labeled voxels by applying a manual brightness threshold that included the IO and its projections and excluded unlabeled background regions (2 camera gray values above background on average). Voxels were placed into categories using thresholds based on their response strengths, as follows. For forward and backward preference (Fig. 4-1*A* *ii*), we considered only the six stimuli with a forward or backward component and defined the following two categories: (1) voxels with a forward motion preference (cos(PD) > 0) and an average dF/*F*_0_ > 0.5 over the three forward stimuli (referred to as forward-selective voxels; Fig. 4-1*A* *ii*, green) and (2) voxels with a backward motion preference (cos(PD) < 0) and an average dF/*F*_0_ > 0.25 over the three backward stimuli (backward selective; Fig. 4-1*A* *i-ii*, magenta). The difference in manually selected thresholds reflects the observed difference in amplitude between forward and backward motion responses (Fig. 2*D*). This choice was made to minimize spread of detected voxels outside the somatic regions, which would reduce measures of overlap, in order to give the fairest consideration to each hypothesis. To compare ipsiversive and contraversive responses (Fig. 4*D*), we only considered left, right, forward-left, and forward-right stimuli, and selected voxels with an average dF/*F*_0_ > 0.25 in either the left or the right directions, grouped according to which direction gave the stronger response. Rotation-selective voxels (Fig. 4-1*A* *i*, green) were selected based on a dF/*F*_0_ > 0.75 to one rotational direction and <0.25 to the other. To display 2D projected maps of the response distributions, we summed the number of respective voxels in each category. Since the number of voxels in the IO was much larger than the sparser cerebellar projections, the top and bottom halves of each image, which corresponded to CF projections and IO neurons’ soma, respectively, were normalized independently to the maximum observed value.

#### Quantification of overlap

To quantify the overlap between functional and anatomical classes we calculated a matching index (MI, Table 1) according to the following protocol. First, the light-sheet and single-cell data was registered to a common anatomical reference, and data were pooled from the left and right side by reflecting across the midline. Distributions were analyzed in two dimensions using a *z*-projection for the cerebellar cortex, and a lateral projection for the IO, since these were the views where the two neuronal types were clearly separated. The location of axon terminals was estimated by taking the endpoints of the neuron skeletons using the “endpoint” option of the MATLAB *bwmorph* function. We estimated the probability distribution for both the anatomical and functional data using a kernel density estimate based on a convolution of the raw data with a 10 pixel (7.8 micron) S.D. Gaussian kernel. The matching score (MS) between an individual anatomical or functional class was taken as the 2D integral of the minimum of the two probability distributions, which will be a value between 0 (no region of overlap) and 1 (perfect overlap). The MI was then calculated as the average MS for one anatomy/function pairing minus the average MS for the opposite pairing, giving a value that can range from −1 to 1, with the sign indicating which pairing gives the best match (1-1 and 2-2 or 2-1 and 1-2). To estimate the statistical significance of the MI, we used the following bootstrapping procedure. We first formulated a null hypothesis that the observed overlap results from random chance. A null distribution corresponding to this hypothesis was generated by calculating the MI for 10,000 random pairs of neuron classes generated by resampling from the data with replacement. The null hypothesis was tested against a one-tailed alternative that the MI was higher than expected from chance with 5% significance level, with Bonferroni correction applied considering the six comparisons (three functional mappings times two brain regions: IO and cerebellum). A match was therefore regarded as significant if the MI was greater than the 99.16th percentile of the null distribution (100, 5/6 = 99.17) for both the soma and axon regions.

**Table 1.** Quantification of functional/anatomical overlap

	MI between anatomical (unipolar/multipolar) and functional mapping		MI significance thresholds	
	Neuropil	Soma	Neuropil	Soma
Ipsi-/contraversive (Fig. 4*F*)	0.28 (*)	0.30 (*)	0.17	0.20
Rotation/backward (Fig. 4-1*A*)	−0.36 (n. s.)	0.21 (*)	0.20	0.17
Forward/backward (Fig. 4-1*B*)	0.05 (n. s.)	0.04 (n. s.)	0.09	0.04

Functional mapping based on responses to ipsi- VS contraversive motion, but not on rotational VS backward and forward VS backward motion, significantly matches the morpho-anatomical mapping.

Matching index indicates how well a given functional mapping overlaps with morpho-anatomical mapping of unipolar/multipolar neurons, independently for the IO neuron’s soma and their projectiles in the cerebellum (see Materials and Methods for details). Its absolute value can range from 0 (no overlap) to 1 (perfect overlap), and its sign indicates which pairs are matched. For example, the negative MI for Rotation / backward mapping in the neuropil area indicates that, in contrast to our expectation, the rotation-selective voxels in the cerebellum overlap better with the projections of multipolar neurons. To calculate the statistical significance of the MIs, we formulated a null hypothesis that the measured MI values are observed by chance. If so, random shuffling of the data should not change the MI in a consistent way. We therefore generated the corresponding null distributions of shuffled data and computed their 99.17*^th^* percentiles as MI significance thresholds (one-tailed alternative, 5% significance level, Bonferroni-corrected for six comparisons: 100—5/6 = 99.17). If an actual MI is higher than the threshold, it is less than 5% that such high MI is observed by mere chance (indicated by asterisks). Only the ipsi- VS contraversive functional mapping significantly matched the anatomical mapping in both the IO and the cerebellum.

### Anatomical registration

To represent functional and anatomical data acquired from different larvae within a common coordinate system, all datasets were registered to one of the two common reference stacks. Anatomical registration was performed using affine volumetric transformation computed by the CMTK (Rohlfing and Maurer, 2003).

The first common reference stack included the IO and the cerebellum to cover the location of the IO cell bodies and their projections (IO-CF reference, e.g., Fig. 1*B*). To obtain the IO-CF reference stack, reference channels of the confocal stacks acquired for single cell labeling (see above) were registered to one of these stacks and then averaged. We used the IO-CF reference to register not only the single-cell anatomical data (*N* = 39 fish) but also all light-sheet functional imaging data (*N* = 28 fish). Some light-sheet datasets could not be registered well to the selected template, and, in these cases, we registered these to the other individual fish that were well registered, and chose the best match. This process was repeated until all functional imaging data was registered to the IO-CF reference.

The second reference stack represented an IO subsection of the IO-CF reference (IO reference). It was used for anatomical registration of the two-photon functional imaging datasets, where CFs were not imaged (*N* = 18 larvae: 12 fish from Experiment 2 and 6 fish from Experiment 3).

Finally, to represent the anatomical organization of the data in the context of the whole larval zebrafish brain (e.g., Fig. 1*A*), the IO-CF reference was registered to a whole brain reference stack that was previously acquired in the Portugues laboratory by co-registration of 23 confocal *z*-stacks of zebrafish brains with pan-neuronal expression of GCaMP6f (*elavl3:GCaMP6f*; *a12200Tg*) (Wolf et al., 2017). To perform this registration, a confocal stack of *elavl3:GCaMP6 s* (*a13203Tg*); *hspGFFDMC28C* (*rk8Tg*); *UAS:mCherry*) (Kim et al., 2017) was used as a bridge, as it contained both pan-neuronal signal (GCaMP6 s) and IO-CF-specific signal (mCherry) in different channels.

## Results

### IO neurons can be divided into distinct morpho-anatomical types

We first aimed to characterize the morpho-anatomical organization of the IO neurons using single-cell labeling. To label IO neurons we used the transgenic hspGFFDMC28C GAL4 driver line (Takeuchi et al., 2015), whose pattern of expression within the zebrafish olivo-cerebellar system is mainly limited to IO neurons and their CFs (Fig. 1*A*), as also shown by previous immunohistochemical and anatomical characterization (Takeuchi et al., 2015). By reconstructing morphologies of single IO neurons using electroporation or sparse genetic labeling (Fig. 1*B,C*), we confirmed that all IO neurons project contralaterally (Fig. 1*D*). By analyzing the dendritic morphology of labeled IO neurons, we found that they can be divided into at least two classes. Neurons of one type have a single dendritic tree that arborizes along the midline of the brain. We refer to such neurons as unipolar (*N* = 16 neurons; Fig. 1*E*, Fig. 1-1*A*, green). Neurons of the second type have dendrites arborizing on both the medial and lateral sides of the IO and are referred to as multipolar (*N* = 19; Fig. 1*E* and Fig. 1-1*A*, magenta). Based on co-registration to a common reference brain, we found that these two types of neurons are located in different regions of the IO and have different projection patterns in the cerebellum: unipolar neurons are mainly located in the ventral–rostral area of the IO and project to the dorsal-medial area of the cerebellum, whereas multipolar neurons are located in the caudal–dorsal part of the IO and project to the ventral–lateral cerebellum (Fig. 1*F*). About one third of labeled neurons (*N* = 18) had dendritic morphology that could not clearly be placed in one of the two categories (Fig. 1-1*A*) and their location and projection patterns lacked any consistent structure (Fig. 1-1*B*). Such ambiguous neurons may represent different cell types in the IO that are less commonly labeled, or neurons that were immature or damaged by the electroporation. These findings show that at least two distinct types of IO neurons can be distinguished based on their dendritic morphology, projection patterns and location within the IO.

### The majority of IO neurons are sensitive to translational and rotational motion, direction selective and spatially organized

We next aimed to characterize the functional organization of the IO. To this end, we used the same GAL4 line to drive expression of a GCaMP calcium indicator (GCaMP6fEF05, see Materials and Methods) specifically in the IO neurons. Using two-photon calcium imaging, we recorded activity of the IO neurons in 6–7 dpf larvae (Fig. 2*A*) in response to translational and rotational motion. Such stimuli are known to elicit distinct behavioral responses in larval zebrafish (OMR and OKR, respectively) that are associated with activity in different areas of the cerebellum (medial and lateral, respectively; Matsui et al., 2014). The stimulus set consisted of drifting gratings in 8 different directions, and rotational motion of a radial “windmill” pattern in both CW and CCW directions (Fig. 2*B*).

We first asked, how many of the IO neurons respond to such stimulation. To answer this, we measured their fluorescence before and during each stimulus presentation and selected those neurons that responded to at least one stimulus (see Materials and Methods for the response criterion used in this study). Such neurons were referred to as active, and the majority of the imaged IO neurons fell into this category (891/1,106 neurons, 81%). Next, to determine whether IO neurons responded to moving stimuli in a direction-selective manner, we computed the direction selectivity index (DSI) for each active neuron, as the vector sum of mean responses to all directions divided by the total response for that neuron. This index ranges from 0 (equal responses to all directions) to 1 (response only to one direction). We found that, for 608 active neurons (68% af active neurons), this index was significantly higher than expected from random nondirection-selective responses (see Materials and Methods). We referred to such neurons as direction-selective. Based on responses to rotational stimuli, we divided all DS neurons into 4 classes: neurons could be described as direction selective only (DS) if they responded exclusively to the translational motion stimuli, or DS + CW or DS + CCW if they also responded to clockwise or counter-clockwise rotation, respectively. Some direction-selective neurons responded to rotational motion in a nonselective manner DS + CW + CCW (Fig. 2*C,D*).

Although we could find neurons tuned to all directions within each of these four classes (Fig. 2*D*), the distribution of PDs was different between classes (Fig. 2*E*). Direction-selective neurons have an overall preference for forward over backward motion (Fig. 2*E*, all direction-selective neurons). Neurons that only responded to translational motion (Fig. 2*E*, DS) had a more even distribution of forward and backward PDs. On the other hand, direction-selective neurons that also responded to rotational motion were mostly tuned to forward motion (Fig. 2*E*, DS + CW, DS + CCW and DS + CW + CCW). This is also clear if we look at the probability distribution of the cosines of the PD for different classes, with cosine close to 1 corresponding to forward and −1 to backward preference (Fig. 2*E*, right). Taken together, these results show that the majority of IO neurons are driven by visual motion stimuli and that the majority of these visually driven neurons are direction selective. The distribution of PDs has a large peak around forward motion, and a smaller peak for backward directed motion.

We next asked whether observed response types are spatially organized within the IO, as we observed for the two aforementioned morphological classes. To this end, we registered all anatomical stacks of imaged fish to a common anatomical IO reference (see Materials and Methods). We found that DS neurons were indeed spatially organized: neurons tuned to forward motion were located more rostrally (Fig. 2*F*, green distribution), while backward-selective neurons had a more caudal position within the IO (Fig. 2*F*, magenta distribution). These neurons also had a different left–right tuning bias depending on which side of the IO they were located. Forward-selective neurons showed an ipsiversive bias in their PD, with more neurons in the left IO having a leftward bias (Fig. 2*F*, blue distribution), while a rightward bias was found in the right IO (Fig. 2*F*, orange distribution). Interestingly, the more caudal backward-selective neurons had the opposite trend, showing a contraversive bias in their PD. Neurons with strong responses to rotational motion showed a very strong lateralization, with responses to CW rotation found predominantly in the rostral right IO, and CCW-responding neurons concentrated in the rostral left side (Fig. 2*G*).

In summary, these results suggest a majority of the IO neurons respond to moving stimuli in a direction-selective manner, with tuning to forward motion being the most typical. Furthermore, direction-selective IO neurons are spatially organized, with rostral neurons being more forward-selective and sensitive to rotational motion, and neurons in the caudal region more tuned to backward translational motion.

### The majority of IO neurons receive input from both eyes with a contralateral bias

In the larval zebrafish, all retinal ganglion neuron axons project contralaterally (Burrill and Easter, 1994). In order to understand how the responses described above could be constructed from these monocular visual inputs, we looked at the integration of visual information in IO neurons by presenting both monocular and binocular visual stimuli (Fig. 3*A*,*B*).

To quantify whether a responsive IO neuron receives sensory information from both eyes or from only one, we defined a monocular index for each neuron. It was calculated as the difference between responses to stimuli presented to the contralateral and ipsilateral eyes (averaged across repetitions and directions), divided by the sum of these responses (see Materials and Methods). This index ranges from −1, which indicates that a neuron responded only to ipsilateral eye stimulation (ipsi-monocular neuron), through 0, indicating that a neuron equally responded to stimulation of both eyes (binocular), to +1, indicating a neuron with input solely from the contralateral eye (contra-monocular). We found that some neurons had a monocular bias, meaning that they responded more strongly to stimuli presented to one eye than to the other one (see Fig. 3*C* *i*-*ii* for examples of monocular neurons; Fig. 3*D* for distribution of monocular indices). However, most neurons were binocular (Fig. 3*D*; for examples of binocular neurons see Fig. 3*C* *iii-vi*). Furthermore, IO neurons tend to have a contralateral bias, i.e. being more sensitive to stimulation of the contralateral eye. For example, the neuron shown in Figure 3*C* *i* responded more strongly to the left eye stimulation, and it was located on the right (Fig. 3*E*); the neuron shown in Figure 3*C* *ii* shows the equivalent pattern for a neuron on the left side. This contralateral bias is also evident from the shift of the monocular indices distribution towards 1 (Fig. 3*D*). The monocular bias was evenly distributed throughout the IO without an apparent spatial organization (Fig. 3*E*).

We next asked which properties of the IO neurons could account for the difference between neurons that responded to both translational and rotational motion (Fig. 2*C,D*, DS + CW, DS + CCW, DS + CW + CCW) and neurons that only responded to translational motion (Fig. 2*C,D*, DS). We hypothesized that, in contrast to neurons that did not respond to any rotational motion, neurons that did could have i) different strengths of inputs from the left and right eyes, and/or ii) different PDs within left and right visual fields.

To test the first hypothesis, that differences in input strength are responsible for sensitivity to rotational motion, we tested whether the magnitude of the monocular index for each neuron correlated with the strength of response to rotational stimuli presented binocularly. Although some of the neurons with high monocular index did respond to rotational stimuli (Fig. 3*C* *i-ii*), we did not observe such correlation on the population level (Fig. 3-1*A*, left). Therefore, differences in monocular bias cannot by themselves explain the variation in sensitivity to rotation.

Binocular rotational motion provides opposing directions of motion to each eye (e.g., CW rotation is forward on the left and backward on the right). To test whether responses to rotational stimuli could be explained by different PDs within the left and right visual fields, we calculated PDs of binocular IO neurons while only the left or right visual field of the larvae was stimulated (left PD and right PD). We observed that the majority of such neurons had similar left and right PDs (Fig. 3-1*B*, bottom left and upper right quadrants, and example neurons in Fig. 3*C* *v-vi*), and, as expected, their binocular PDs were similar to the monocular PDs (i.e., they are mostly green and magenta, respectively). However, we found that a substantial fraction of IO neurons (*N* = 91 (26.8%) neurons out of 340 direction-selective neurons) had opposing PDs between left and right visual fields (Fig. 3-1*A* right, Fig. 3-1*B* bottom right and upper left quadrants, and example neurons in Fig. 3*C* *iii, iv*). Such neurons also showed strongest sensitivity to rotational motion presented binocularly (Fig. 3-1*C*).

Interestingly, the majority of such neurons with opposing PDs between the two eyes were strongly tuned to forward motion when stimulated binocularly, despite one of the two monocular inputs being tuned to backward motion (Fig. 3-1*B*, bottom right and upper left quadrants). This is consistent with the directional tuning properties of DS + CW and DS + CCW from the binocular experiment, which were tuned to forward motion and also responded to rotation (Fig. 2*E*). This suggests the presence of inhibition driven by backward motion on the forward-preferring side, which results in binocular responses that are not the simple sum of monocular inputs (see example neurons in Fig. 3*C* *iii-iv*).

Looking at the distribution of binocular neurons with opposing or similar monocular PDs, we found that they were spatially organized within the IO (Fig. 3-1*D*), with a pattern consistent with the spatial organization observed in the previous experiment (Fig. 2*F,G*). Neurons with forward monocular PDs were found more rostrally (green distribution in Fig. 2*F* and Fig. 3-1*D*), while those with backward PDs had a more caudal location (magenta distribution in Fig. 2*F* and Fig. 3-1*D*). Neurons with opposing PDs occupied a rostral and lateral position with a left-right bias depending on which of the two monocular inputs was tuned to forward motion. Neurons with left forward tuning (i.e., those that typically respond to CW rotation) were concentrated in the rostral right IO (Fig. 2*G* and red population in Fig. 3-1*D*), whereas neurons with right forward tuning (CCW-sensitive) were more predominant in the rostral left IO (Fig. 2*G* and blue population in Fig. 3-1*D*).

In summary, most IO neurons receive binocular input, in general a stronger one from the contralateral eye. A number of IO neurons were specifically tuned to one of the directions of rotational motion and had opposing PDs between the eyes, i.e. neurons strongly responded to CW or CCW rotation if their left or right respective field was tuned to forward motion, respectively. When larvae were stimulated with translational motion binocularly, these neurons typically preferred forward motion and did not respond to translational motion to the back, even though either their left or right visual receptive field was tuned to backwards motion. This suggests that binocular neurons do not simply sum their monocular inputs, but that they integrate these nonlinearly, in order to compute a behaviorally relevant stimulus feature. Finally, binocular neurons were spatially organized within the IO according to their function.

### Functional organization of the IO maps onto its morpho-anatomical organization

From our morpho-anatomical investigation, we found that two regions could be identified within the IO, referred to as caudal and rostral, that contain neurons with different dendritic morphologies and projection patterns (Fig. 1). We were interested in whether this spatial separation of morpho-anatomical types mapped onto the observed functional organization (Fig. 2*F,G*; Fig. 3-1*D*). Since one of the predictable features of the neuron’s morphological type was its projection pattern within the cerebellum (Fig. 1*F*), we performed volumetric light-sheet imaging to simultaneously record calcium activity not only from IO neurons’ soma but also from their axon terminals within the cerebellum (Fig. 4*A*).

As in the previous experiments with binocular visual stimulation (Fig. 2), the protocol consisted of translational gratings moving in eight different directions, in a randomized order, followed by rotational motion in CW and CCW directions. In addition, after the two rotational stimuli we added two more: converging and diverging stimuli (Fig. 4*B*). Active voxels were detected across 28 fish, and anatomically registered to the same reference space as the single neuron morphology data. Consistently with the previous two-photon data (Fig. 2*F*), direction-selective activity was observed throughout the IO, and, in each fish, voxels in the cerebellum were found with similar tunings to those found in the contralateral IO. The pooled data showed the spatial distribution expected from our previous results. Thus, forward-selective voxels clustered in the rostral and medial regions of the IO (Fig. 4*C*, green), and rotation-selective voxels found rostrolateral to these (Fig. 4*C*, red). Backward-selective voxels were located more caudally (Fig. 4*C*, blue) (see also Extended Data Movies 1, 2, and 3).

10.1523/JNEUROSCI.2352-21.2023.video.1 Movie 1 3D rendering of distribution of rotation-selective voxels in light-sheet imaging data shown in Supplementary Figure 4Ai with those preferring clockwise motion shown in green and counterclockwise motion in magenta. Download Movie 1, MP4 file.

10.1523/JNEUROSCI.2352-21.2023.video.2 Movie 2 3D rendering of distribution of forward (green) and backward (purple) preferring voxels in light-sheet imaging data shown in Supplementary Figure 4Aii. Download Movie 2, MP4 file.

10.1523/JNEUROSCI.2352-21.2023.video.3 Movie 3 3D rendering of distribution of rotation-selective voxels in light-sheet imaging data shown in Supplementary Figure 4Aiii with those preferring leftward motion shown in green and rightward motion in magenta. Download Movie 3, MP4 file.

After confirming that spatial distribution of functional properties is consistent with our two-photon data, we asked whether these properties map onto the unipolar/multipolar morphological classes described in Figure 1. To quantify how well morpho-anatomical mapping overlaps with a given functional mapping, we defined a MI (see Materials and Methods) (Table 1). Its absolute value can range from 0 (no overlap) to 1 (perfect overlap, i.e. anatomy and function can be perfectly predicted from each other). The sign of the MI depends on which pairs of compared classes are matched.

We first focused on the rotation- and backward-selective voxels. In our two-photon data, we found that neurons sensitive to rotational motion were concentrated in the rostral IO (Fig. 2*G*, Fig. 3-1*D*), whereas neurons tuned to backward translational motion were located more caudally (Fig. 2*F*, magenta distribution; Fig. 3-1*D*). Since unipolar and multipolar neurons showed rostral-caudal spatial distribution consistent with that pattern (Fig. 1*F*), we hypothesized that rotation- and backward-selective voxels may correspond to unipolar and multipolar neurons, respectively. Analysis of the overlap within the IO was consistent with this hypothesis: location of rotation- and backward-selective voxels overlapped significantly with the location of unipolar and multipolar neurons; somata, respectively (Fig. 4-1*B* *i*, Table 1). Since unipolar neurons project more medially in the cerebellum than multipolar ones (Fig. 1*F*), if this hypothesis is true, rotation-selective voxels should also project more medially than the backward-selective ones. However, analyzing the 3D positions in the cerebellar projection fields of these particular functional groups, we found that the opposite was true: the terminals of rotation-selective neurons occupied more lateral positions in the cerebellum than terminals of backwards-selective neurons (Fig. 4-1*A* *i,* different sign of MI in Table 1). Therefore, spatial distribution of rotation- and backward-selective voxels in both the IO and cerebellum was inconsistent with the hypothesis that these functional types correspond to the morpho-anatomical types.

We next focused on forward- versus backward-selective voxels and hypothesized that these functional groups may correspond to unipolar and multipolar neurons, respectively. The forward-selective voxels overlapped well with the distribution of somata of unipolar and some multipolar neurons (Fig. 4-1*B* *ii*), however this overlap was not statistically significant (Table 1). Furthermore, forward-selective terminals were found with a wide distribution extending both medial and lateral with respect to the backward-preferring domain (Fig. 4-1*A* *ii*), also not consistent with a simple correspondence with unipolar or multipolar neurons (Table 1).

We therefore did not find evidence for spatial correspondence between the two morpho-anatomical classes and both rotation- versus backward-selective and forward- versus backward-selective functional types. We next asked if there was another functional mapping that more closely matched the morphological organization. Apart from spatial organization of rotational responses and forward and backward PDs, in our two-photon imaging experiments we also observed a left-right bias in the distribution of PDs, with rostral and caudal neurons tuned to ipsi- and contraversive motion, respectively (Fig. 2*F*, blue and orange distributions). Based on this observation, we hypothesized that ipsiversive- and contraversive-selective neurons may correspond to unipolar and multipolar neurons. We found that the functional organization in our light-sheet data was consistent with this hypothesis. Within the large population of voxels that responded to forward motion, voxels responding to ipsiversive motion were sitting more rostral to those that preferred contraversive motion (Fig. 4*D* *ii*). Furthermore, the terminals of the more rostral domain were located in the cerebellum more medially than those of the caudal group (assuming a crossed projection (Fig. 1*D*)), consistent with the observed projection patterns of unipolar and multipolar IO neurons (Fig. 4*D* *i*). Superimposing these two functional domains on the arborizations of IO neurons (Fig. 4*E* *i*) and the distribution of their somata (Fig. 4*E* *ii*) showed a significant correspondence between these anatomical and functional divisions (Fig. 4*F* and Table 1).

These results suggest the existence of at least four spatial divisions within the olivo-cerebellar pathway, solely based on sensitivity to whole-field motion, with characteristic soma distributions and projection patterns. While the organization based on ipsiversive/contraversive sensitivity may correspond to the anatomical distinction between unipolar and multipolar IO neurons, the rotation-, forward- and backward-selective groups may have been less frequently labeled in our morphological dataset and/or categorized as “ambiguous”. Such nonclassified neurons which may represent additional morphological classes were not described in this study (Fig. 1-1).

Taken together, our data show that morphologically different classes of IO neurons are spatially organized within the IO and in their projection patterns and may have different functional properties, contributing to the division of cerebellum into different functional compartments.

## Discussion

In this study, we show that IO neurons of larval zebrafish can be divided into at least two types based on their dendritic morphology, location and cerebellar projection pattern. Moreover, these anatomically defined classes of IO neurons appear to correspond to distinct functional types, and project to nonoverlapping regions of the cerebellum associated with distinct visually driven behaviors such as the OMR and OKR (Matsui et al., 2014).

Neurons here referred to as unipolar are located in the rostral IO and have unipolar dendrites arborizing along the midline. Multipolar neurons are located in the caudal IO and have bi- or tri-polar dendritic trees arborizing on the medial and lateral sides of the IO (Fig. 1*E,F*). Beyond dendritic morphology, the two classes of IO neurons we describe exhibited distinct projection patterns, with multipolar neurons projecting to the medial–lateral cerebellum and unipolar neurons projecting their axons to the medial cerebellum (Fig. 1*F*). In mammals, IO neurons are electrically coupled (Llinas et al., 1974; Leznik and Llinás, 2005) and have been shown to be very heterogeneous in their dendritic morphology, encompassing a continuum of “curly” and “straight” morphology types (Vrieler et al., 2019). According to their dendritic tree orientation and soma localization, it has been suggested that neurons within the IO network are organized into areas of stronger or weaker coupling but with no clear clustering (Vrieler et al., 2019). One possibility is that the multipolar IO neurons we observed here could serve as “link neurons” to electrically couple multiple neighboring cells. The dendritic morphology of these neurons also makes them good candidates for integration of signals from different inputs and possibly convergence of multimodal signals, similar to cerebellar granule cells (Ishikawa et al., 2015; Knogler et al., 2017).

We found that the majority of IO neurons that we labeled in larval zebrafish were very sensitive to visual stimuli with strong direction selectivity (Fig. 2*D*). Consistent with previous reports describing complex spikes in Purkinje cells (Knogler et al., 2019; Markov et al., 2021), IO activity was generally phase-locked to motion onset (Fig. 3*C*), suggesting a role of these neurons as “sensors”, rather than “integrators” of sensory evidence (Bahl and Engert, 2020; Dragomir et al., 2020; Markov et al., 2021). Inferior olive neurons’ preferred directions covered the full range of directions presented (Fig. 2*D*), but responses tuned to forward or backward directions were relatively overrepresented (Fig. 2*E*). We also observed a bias towards forward motion (Fig. 2*D,E*), although previous studies have reported equally well-represented complex spike responses to backward motion onset (Knogler et al., 2019). This could be due to an approximate twenty-fold increase in the number of recorded neurons in our study. It is also possible at this early stage of development that not all IO neuron activity that is observed will result in Purkinje cell complex spikes.

The direction-selective visual responses we observed within the IO were spatially organized, with forward and backward preferred neurons located in a rostral and caudal position, respectively (Fig. 2*F*, green and magenta distributions). We also found that IO direction-selective neurons had different left-right tuning biases depending on their position along the rostral–caudal and left–right axes. Rostral neurons that preferred forward motion had a bias in preferred direction towards the ipsilateral side; whereas the caudal backward-selective neurons had a contralateral bias (Fig. 2*F*, blue and orange distributions). The same was true for neurons sensitive to rotational motion, with CW responses more prominent in the rostral right IO and CCW responses in the rostral left side (Fig. 2*G*). This laterality in rotation preference aligns with the organization of the hindbrain oculomotor centers, which predominantly drive ipsiversive eye movements.

We found that most IO neurons were binocular, i.e., they were sensitive to visual information coming from both the left and right eye (Fig. 3*D*). Although neurons that responded to rotation presented binocularly had opposing preferred directions between the eyes, these neurons typically preferred forward motion, and did not respond to backward motion, when stimulated with translational motion binocularly (Fig. 3-1*B*). These properties are consistent with recordings from neurons in the dorsal cap of the IO in rabbits, where binocular neurons displayed dominant input from one eye, which, for neurons sensitive to vertical axis rotation, was the contralateral eye (Leonard et al., 1988).

It would be interesting to investigate the nature of inputs to the IO and if and how these are in turn topographically organized, especially considering recent work that has shown a topographic organization of translational motion sensitivity in the zebrafish pretectum (Zhang et al., 2022). Although IO inputs have not been mapped in zebrafish specifically, both mammals and other teleosts have a population of IO neurons that receives input from the accessory optic system or the pretectum, respectively (Brown et al., 1977; Xue et al., 2008; Yáñez et al., 2018). Many pretectal neurons are monocular but mechanisms for binocular integration of the optic flow have been proposed to be computed at the pretectum circuit level (Kubo et al., 2014; Naumann et al., 2016; Kramer et al., 2019; Wang et al., 2019), and a topographic organization of PD in a population of binocular neurons in the pretectum was recently described (Yildizoglu et al., 2020, Zhang et al., 2022).

In this study, IO neurons convey whole-field visual motion signals. In zebrafish, the IO has been implicated in modulating several aspects of whole-field motion responses, including gain adaptation, calibration of the feedback controller, and positional homeostasis (Ahrens et al., 2012; Markov et al., 2021; Yang et al., 2022). During swimming, optic flow is also integrated with other signals to guide behavior, such as lateral line and vestibular inputs (Olszewski et al., 2012; Suli et al., 2012; Ehrlich and Schoppik, 2017; Oteiza et al., 2017). In addition to visual information, the zebrafish IO may also receive mechanosensory signals from the lateral line, trigeminal and spinal somatosensory and vestibular signals conveyed from pretectum, tectum, red nucleus and octaval nucleus inputs (Xue et al., 2008).

Vestibular responses have been reported in the larval zebrafish IO, independently of visual input (Migault et al., 2018). These responses are also direction-selective and spatially organized in a manner that is consistent with our results, as in both cases, responses correspond to ipsiversive eye movements. In several species, rotation-selective neurons have preferred axes that align with the axes of rotation sensed by the semicircular canals (Leonard et al., 1988), although this appears to vary across fish species (Masseck and Hoffmann, 2008, 2009). Our stimulus presentation geometry does not allow us to assess this directly in the zebrafish larvae, although we also note that the semicircular canals are too small to be functional at this age (Bever and Fekete, 2002; Beck et al., 2004).

In theories of cerebellar function, CF projections from the IO are thought to be important for calibrating motor output by providing instructive signals for learning (Marr, 1969; Albus, 1971; Ito et al., 1982, Silva et al., 2022), as well as for establishing discrete functional modules (Apps and Garwicz, 2005; Herzfeld et al., 2015). In zebrafish, functional imaging and electrophysiology studies have associated the medial and lateral cerebellum regions to the OMR and OKR, respectively (Matsui et al., 2014; Knogler et al., 2019). Here, we find that the spatial separation of different functional types exists already in the IO. This appears to be a conserved organizational feature in vertebrates, as, for example, the ventral uvula of the cerebellum in pigeons has also been shown to be organized into domains sensitive to different optic flow patterns, and receives topographically organized projections from the IO (Craciun et al., 2018). Elucidating the functional organization of the olivo-cerebellar pathway will help us to understand the nature of information processing within this highly conserved circuit.
