# Supplementary figures and images for: Structural and Functional Organization of Visual Responses in the Inferior Olive of Larval Zebrafish
Source: J Neurosci. 2024 Feb 21;44(8):e2352212023. doi: 10.1523/JNEUROSCI.2352-21.2023 (PMC10883660; doi:10.1523/JNEUROSCI.2352-21.2023)

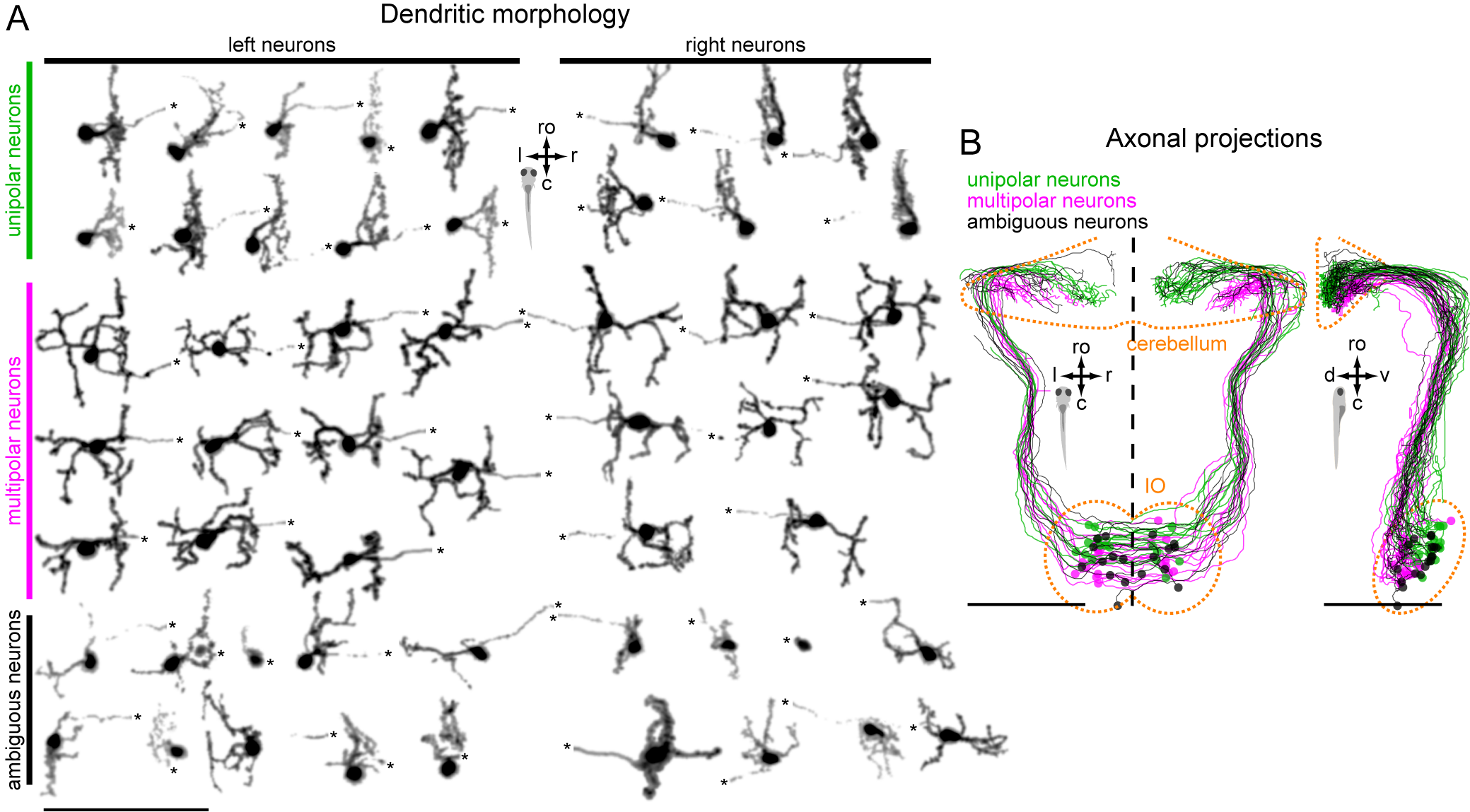

Supplement: Figure 1-1 — The complete dataset of single-labeled IO neurons A. Dendritic morphology of all labeled IO neurons divided by their morphological class (green, 16 unipolar neurons, magenta, 19 multipolar neurons, black, 18 ambiguous neurons). Asterisks indicate axons. B. Axon reconstruction of all labeled neurons; ro, rostral direction, l, left, r, right, c, caudal, d, dorsal, v, ventral; scale bars, 100 µm. Download Figure 1-1, TIF file. [file jneuro-44-e2352212023-s001.tif]

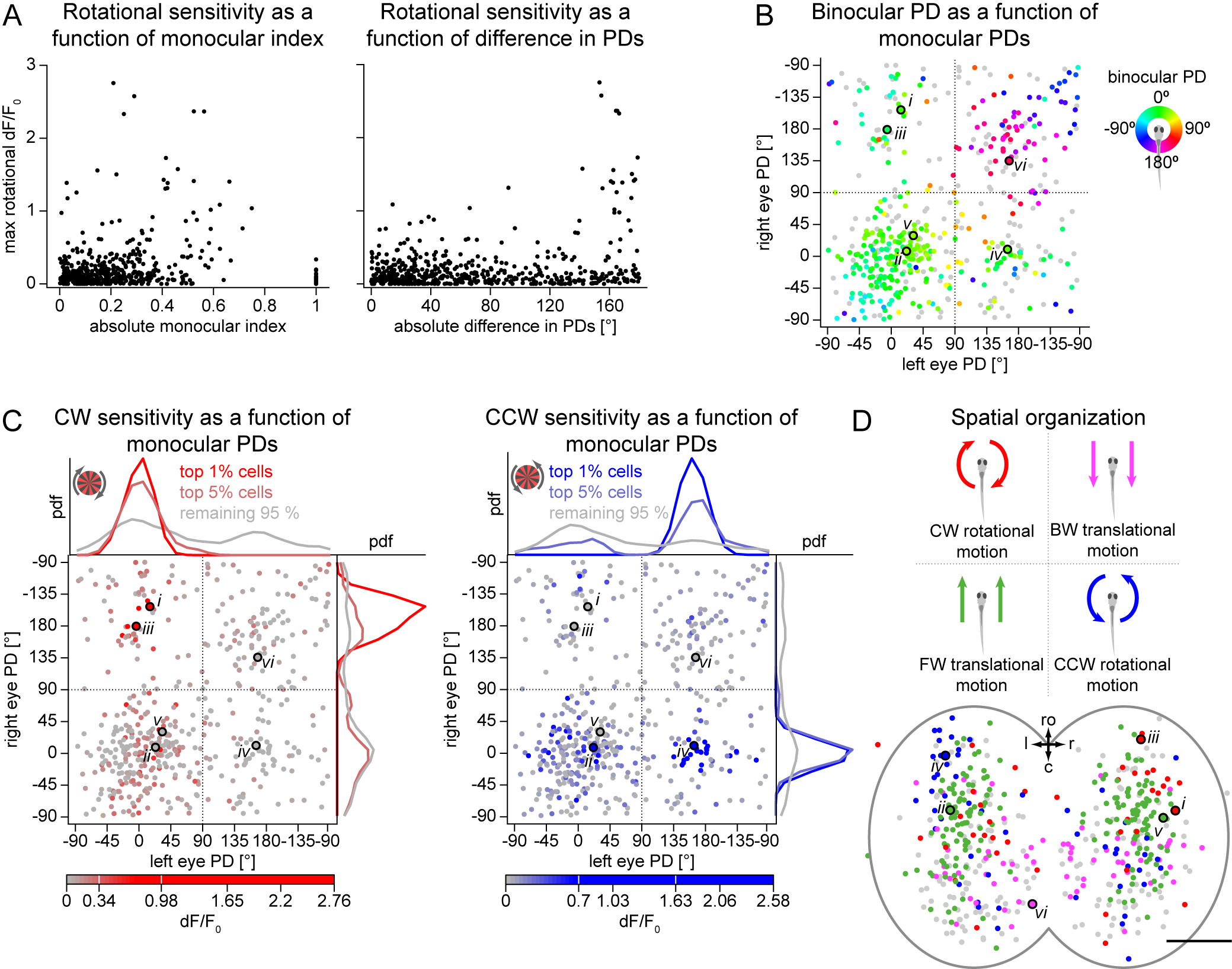

Supplement: Figure 3-1 — Rotation-sensitive IO neurons often receive inputs with opposing PDs and occupy distinct regions within the IO A. Left, maximal response to rotational stimuli as a function of the absolute monocular index of each neuron (left) and of absolute difference in the PDs of the left and right visual fields (right). B. Binocular PD as a function of left and right eye PDs, color-coded as in color wheel on top. Gray dots represent non direction-selective cells. In B, C, and D: small italic roman numbers indicate example neurons shown in Figure 23. C. Responses to binocular CW rotation (left) and CCW rotation (right) as a function of left and right PDs. D. Spatial organization of the IO neurons, color-coded based on their monocular PDs. Shown in red and blue are neurons with opposing PDs (upper left and bottom right quadrants in B and C). Green and magenta indicate neurons with similar PDs (green: forward monocular PDs, bottom left quadrant; magenta: backward PDs, upper right quadrants). Ro, rostral direction, l, left, r, right, c, caudal; scale bar, 25 µm. Small italic roman numbers indicate the location of neurons shown in C. N = 518 neurons (of which 511 were active) from 6 fish. Download Figure 3-1, TIF file. [file jneuro-44-e2352212023-s002.tif]

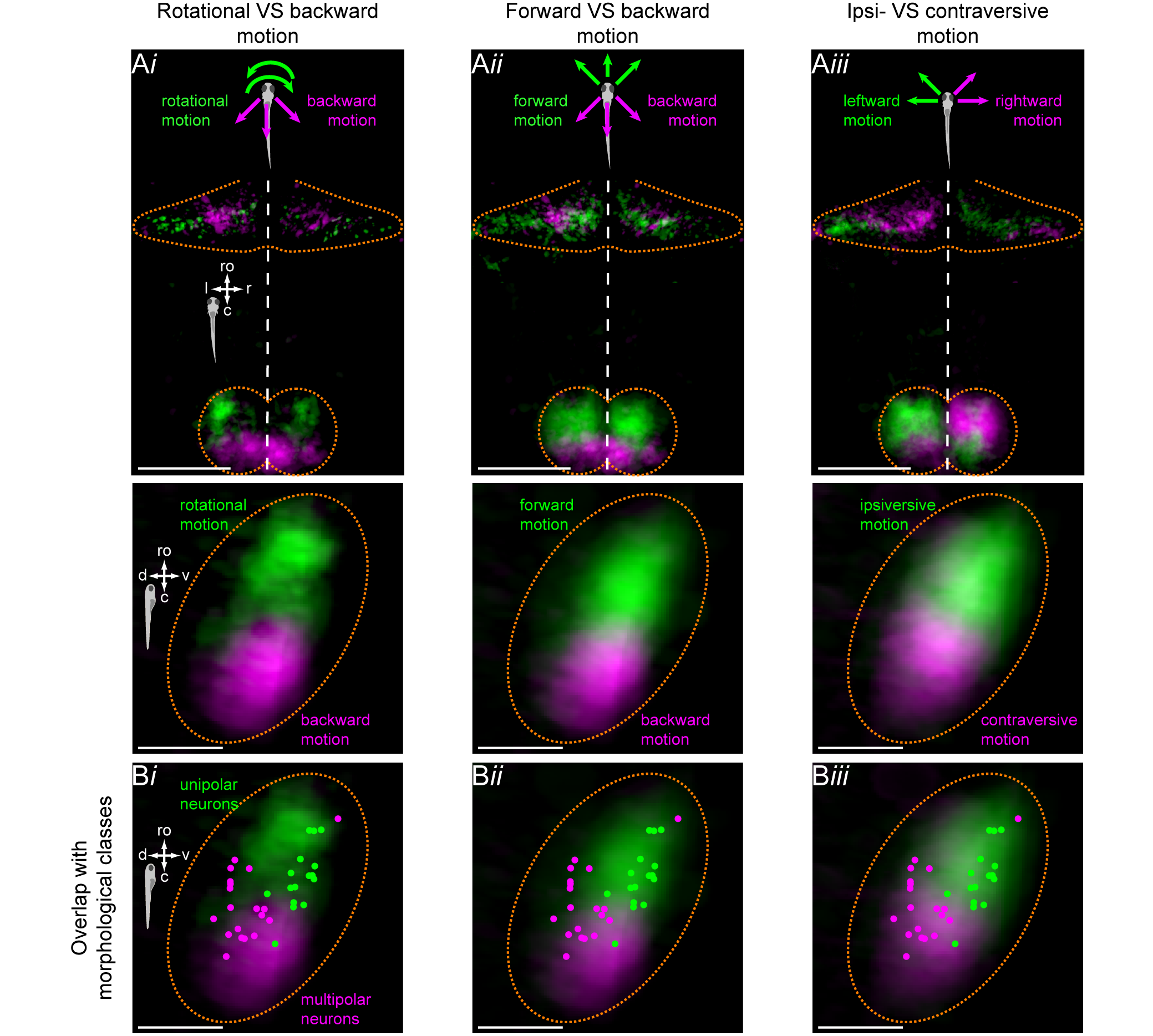

Supplement: Figure 4-1 — Functional mapping based on responses to rotational, forward and backward motion does not correspond to the morpho-anatomical organization of the IO A. Max projections of the distribution of active voxels color-coded using different functional axes: A i, rotational-selective (green) VS backward-selective voxels (magenta); A ii, forward-selective (green) VS backward-selective voxels (magenta). A iii is reproduced from Figure 4D for comparison to other functional mappings. Images on top show max z-projections of the entire imaging field of view that included IO and CFs, bottom images show zoomed-in lateral projections zoomed into the IO. B. Overlap of respective functional maps with morpho-anatomical mapping within the IO. Each dot represents one unipolar or multipolar neuron from Figure 1. See Table 1 for quantification of the overlap. N = 28 fish; ro, rostral direction, l, left, r, right, c, caudal, v, ventral, d, dorsal; scale bars, 100 µm. Download Figure 4-1, TIF file. [file jneuro-44-e2352212023-s003.tif]
